# Teashirt 1 (Tshz1) is essential for the development, survival and function of hypoglossal and phrenic motor neurons in mouse

**DOI:** 10.1242/dev.174045

**Published:** 2019-09-01

**Authors:** Charlotte Chaimowicz, Pierre-Louis Ruffault, Cyril Chéret, Andrew Woehler, Niccolò Zampieri, Gilles Fortin, Alistair N. Garratt, Carmen Birchmeier

**Affiliations:** 1 Developmental Biology/Signal Transduction, Max Delbrück Center for Molecular Medicine (MDC) in the Helmholtz Society, 13125 Berlin, Germany; 2 Systems Biology Imaging, Max Delbrück Center for Molecular Medicine (MDC) in the Helmholtz Society, 13125 Berlin, Germany; 3 Development and Function of Neural Circuits, Max Delbrück Center for Molecular Medicine (MDC) in the Helmholtz Society, 13125 Berlin, Germany; 4 UMR9197, CNRS/Université Paris-Sud, Paris-Saclay Institute of Neuroscience, 1 Avenue de la Terrasse, 91190 Gif-sur-Yvette, France; 5 Institute of Cell Biology and Neurobiology, Charité-Universitätsmedizin Berlin, Virchowweg 6, 10117 Berlin, Germany

**Keywords:** *Tshz1*, Motor neurons, Phrenic motor column, Hypoglossal nucleus, Aerophagia, Breathing, Mouse

## Abstract

Feeding and breathing are essential motor functions and rely on the activity of hypoglossal and phrenic motor neurons that innervate the tongue and diaphragm, respectively. Little is known about the genetic programs that control the development of these neuronal subtypes. The transcription factor Tshz1 is strongly and persistently expressed in developing hypoglossal and phrenic motor neurons. We used conditional mutation of *Tshz1* in the progenitor zone of motor neurons (*Tshz1^MN^*^Δ^) to show that *Tshz1* is essential for survival and function of hypoglossal and phrenic motor neurons. Hypoglossal and phrenic motor neurons are born in correct numbers, but many die between embryonic day 13.5 and 14.5 in *Tshz1^MN^*^Δ^ mutant mice. In addition, innervation and electrophysiological properties of phrenic and hypoglossal motor neurons are altered. Severe feeding and breathing problems accompany this developmental deficit. Although motor neuron survival can be rescued by elimination of the pro-apoptotic factor *Bax*, innervation, feeding and breathing defects persist in *Bax^−/−^; Tshz1^MN^*^Δ^ mutants. We conclude that Tshz1 is an essential transcription factor for the development and physiological function of phrenic and hypoglossal motor neurons.

## Abstract (precis)

**Summary:** Mutation of *Tshz1* in mice impairs the development of hypoglossal and phrenic motor neurons, leading to severe breathing and feeding problems that are ultimately lethal.

## INTRODUCTION

Breathing and feeding are motor behaviors that are essential for the survival of all terrestrial vertebrates. They rely on complex neuronal networks, including motor neurons that are the ‘executive’ components controlling the activity of specific muscle groups (Moore et al., 2014). The diaphragm muscle is essential for inspiration during breathing and is innervated by motor neurons of the phrenic motor column located in the mid-cervical spinal cord. The tongue has crucial functions in feeding and is innervated by the hypoglossal motor neurons in the lower brainstem. The activity of phrenic and hypoglossal motor neurons has to be tightly coordinated to avoid maladaptive outcomes such as the swallowing of air or the blockage of airways (Moore et al., 2014). Hence, hypoglossal and phrenic motor neurons and the circuitry that coordinates their activity are essential for animal survival. Nevertheless, relatively little is known about the formation of the motor neurons that relay breathing and feeding commands.

All motor neurons that innervate skeletal muscle, i.e. somatic motor neurons, derive from ventral progenitor cells expressing the transcription factor *Olig2* and share a common transcriptional program (*Isl1/2*^+^, *Hb9*^+^; *Hb9* is also known as *Mnx1*) during their early initial development that distinguishes them from other neuronal types (Jessell, 2000). However, at later developmental stages they acquire subtype-specific identities that correlate with the muscle groups they innervate (Dasen et al., 2008; De Marco Garcia and Jessell, 2008; Livet et al., 2002). According to their subtype identity, the ventrally derived motor neurons initiate expression of specific sets of transcription factors and either maintain or downregulate *Isl1/2* and *Hb9* (Arber et al., 1999; Briscoe et al., 1999; Pfaff et al., 1996). In addition, their cell bodies cluster, forming motor pools in specific positions along the rostro-caudal and medio-lateral axes of the hindbrain and spinal cord (Bonanomi and Pfaff, 2010; Dasen and Jessell, 2009). Compared with motor neuronal subtypes that innervate different limb muscles, little is known about the genetic programs important for the development of hypoglossal and/or phrenic motor pools.

Genes that specifically affect hypoglossal motor neuron development have not yet been identified. Hypoglossal motor neurons locate near the midline of rhombomeres 7/8 of the hindbrain and express *Isl1/2* and *Hb9*. They are organized topographically according to which tongue muscle they innervate (Aldes, 1990, 1995; Krammer et al., 1979; Lewis et al., 1971; Odutola, 1976). Dorsal hypoglossal motor neurons innervate the retrusor muscles (i.e. extrinsic hyoglossus, extrinsic palatoglossus, intrinsic inferior longitudinalis and intrinsic superior longitudinalis) and express Foxp1 (Chen et al., 2016). In contrast, motor neurons located in the ventral half of the hypoglossal nucleus innervate the protrusor muscles (i.e. extrinsic genioglossus, intrinsic verticalis and intrinsic transversus) and express Pou3f1 (Chen et al., 2016). Finally, motor neurons innervating extrinsic and intrinsic muscles segregate in the lateral and medial hypoglossal nucleus, respectively (Aldes, 1995; Chen et al., 2016; Dobbins and Feldman, 1995; McClung and Goldberg, 2002).

More information is available for phrenic motor neurons that locate to the cervical spinal cord (C3-C5). Previous studies showed that mutations causing respiratory distress often affect the function and/or survival of phrenic motor neurons (Turgeon and Meloche, 2009). These mutations can either affect the survival of all motor neurons, for instance *Hb9*, *ErbB3*, *CLAC-P* (*Col25a1*) or *Fzd3* (Hua et al., 2013; Riethmacher et al., 1997; Tanaka et al., 2014; Yang et al., 2001), or specifically phrenic motor neurons, e.g. *Hoxa5/c5* genes and *Pou3f1* (Bermingham et al., 1996; Machado et al., 2014; Philippidou et al., 2012). Indeed, *Pou3f1* (also known as *Scip* or *Oct6*) is strongly expressed in phrenic neurons, and its ablation in mice leads to disorganization of the phrenic motor column and early postnatal lethality due to breathing failure within the first hours of life (Bermingham et al., 1996). The *Hoxa5/c5* null mutation in motor neurons impairs the expression of phrenic specific markers and the correct localization and survival of phrenic motor neurons, resulting in a complete absence of diaphragm innervation (Philippidou et al., 2012). When phrenic motor neurons are generated from embryonic stem cells, the combined action of Pou3f1 and Hoxa5 is necessary for the acquisition of their identity (Machado et al., 2014).

The transcription factor teashirt zinc finger homeobox 1 (Tshz1) belongs to the evolutionarily conserved family of Teashirt genes that are characterized by the presence of three atypical zinc finger motifs, an N-terminal acidic domain and a binding motif for the co-repressor CtBP (C-terminal binding protein). In addition, mammalian Tshz genes also contain a homeobox domain (Caubit et al., 2000; Fasano et al., 1991; Manfroid et al., 2004; Santos et al., 2010). *Tshz1* is strongly expressed in subtypes of developing and mature neurons of the mouse (Caubit et al., 2005; Kuerbitz et al., 2018; Ragancokova et al., 2014). Mutation of *Tshz1* in mice (*Tshz1^NULL^*) leads to perinatal lethality and severe physiological dysfunctions, such as the inability to feed and respiratory distress, which have been attributed to morphological changes of the oral cavity (Coré et al., 2007). The function of *Tshz1* in motor neurons has not been investigated.

Here, we define the role of *Tshz1* during development of somatic motor neuronal subtypes. We show that *Tshz1* is expressed persistently during development in hypoglossal and phrenic motor neurons, but transiently in other motor neuronal subtypes. We investigated the role of Tshz1 using a conditional *Tshz1* loss-of-function mutation introduced with the *Olig2^Cre^* allele in ventral motor neuron precursors (*Tshz1^MN^*^Δ^). *Tshz1^MN^*^Δ^ mice are born at a normal Mendelian ratio. However, the mutant pups do not feed but instead have air in their stomach and intestine, display respiratory distress and die within the first 10 h of life. Interestingly, two specific motor neuron populations are affected by *Tshz1* loss of function: the hypoglossal nucleus and the phrenic motor column. These motor neurons were generated in appropriate numbers but many subsequently died by apoptosis, leading to a reduction in their numbers at birth and reduced muscle innervation. Elimination of the pro-apoptotic gene *Bax* in *Tshz1^MN^*^Δ^ mice rescued motor neuron numbers. However, neither physiological nor innervation deficits were rescued in the *Bax^−/−^; Tshz1^MN^*^Δ^ mice. Thus, *Tshz1* loss of function impairs motor neuron function as well as survival. We conclude that Tshz1 is an essential transcription factor and coordinates the survival and function of phrenic and hypoglossal motor neurons.

## RESULTS

### *Tshz1* is expressed in specific motor neuron populations

**Fig. 1.**
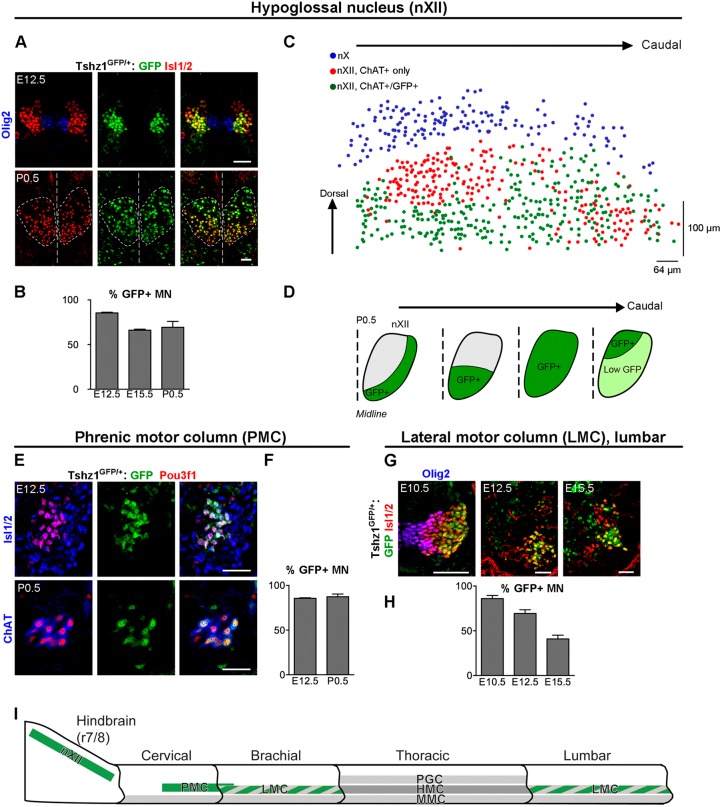
***Tshz1* is expressed in specific motor neuron populations during development.** *Tshz1* expression was assessed by analysis of GFP expression in *Tshz1^GFP/+^* heterozygous mice. (A) Coronal sections of E12.5 and P0.5 hindbrain showed that *Tshz1*-driven GFP expression (green) was detected persistently in developing and mature Isl1/2^+^ hypoglossal motor neurons (red). GFP was not expressed in Olig2^+^ progenitors (blue). At P0.5, the midline and the limits of the hypoglossal nuclei are indicated by dashed lines. (B) Quantification of Isl1/2^+^ hypoglossal motor neurons that co-express GFP at the indicated stages. (C) 3D reconstruction of the hypoglossal motor nucleus (nXII) derived from serial hindbrain sections (P0.5) of a *Tshz1^GFP/+^* animal; the dorsal motor nucleus of the vagus (nX) serves as a landmark. Neuron distributions in each slice analyzed are shown; note that slices are angled for better visualization of the neuronal organization. Blue dots: nX neurons (ChAT^+^); red/green dots: green and red dots display hypoglossal motor neurons (ChAT^+^) that do and do not express GFP, respectively. (D) Restricted *Tshz1* expression in hypoglossal motor neuronal subpopulations at different levels of the rostro-caudal axis. (E) In the cervical spinal cord, GFP (green) was detected in Pou3f1^+^ phrenic motor neurons (red) at E12.5 and P0.5. Isl1/2 (blue, upper panels) or ChAT (blue, lower panels) were used as general motor neuron markers. (F) Quantification of the percentage of phrenic motor neurons expressing GFP at the indicated stages. (G) GFP expression (green) in postmitotic neurons of the LMC (Isl1/2^+^ red); Olig2^+^ progenitors are shown in blue. (H) Quantification of the percentage of motor neurons in the LMC expressing GFP at the indicated stages. (I) Scheme summarizing *Tshz1* expression in different motor neuronal populations. *Tshz1* is expressed persistently in the hypoglossal nucleus (nXII) and PMC (solid green) but is only transiently expressed in LMCs (dashed green). Scale bars: 50 µm. Data are represented as mean±s.d.; *n*=3.

We examined the expression of *Tshz1* in motor neuronal subtypes during development by using heterozygous *Tshz1^GFP^* mice in which the second exon of *Tshz1* was replaced by sequences encoding GFP (Ragancokova et al., 2014). Analysis of *Tshz1*-driven GFP expression in the spinal cord and brainstem showed the presence of GFP in several neuronal types, among them motor neurons. Detailed immunohistochemical analysis using anti-GFP and pan-motor neuron antibodies, such as anti-Isl1/2 or anti-choline acetyltransferase (ChAT), demonstrated the presence of GFP in hypoglossal motor neurons (nXII) from embryonic day (E) 12.5 to postnatal day (P) 0.5 (Fig. 1A). GFP was detected in 85% of the Isl1/2^+^ hypoglossal motor neurons at E12.5, but not in Olig2^+^ progenitors (Fig. 1A,B). GFP expression persisted and was detected in around two-thirds of hypoglossal motor neurons at E15.5 and P0.5. Subsets of GFP^+^ hypoglossal motor neurons co-expressed Foxp1 and Pou3f1 (Fig. S1A,B). The persistent expression of Tshz1 in hypoglossal motor neurons was confirmed by *in situ* hybridization at P0.5 (Fig. S1C). *Tshz1* expression was spatially restricted to subdomains of the hypoglossal nucleus at P0.5, a finding that became apparent in a 3D reconstruction of the hypoglossal nucleus (Fig. 1C; see Fig. S5 for examples of sections used for the reconstruction). The position of hypoglossal motor neurons was digitally reconstructed in three dimensions by assigning Cartesian coordinates to each motor neuron identified in consecutive histological sections (see also Materials and Methods). The position of neurons of the adjacent dorsal nucleus of the vagus nerve (nX) was used as a landmark. In the rostral hypoglossal nucleus, GFP-expressing motor neurons were only detected ventro-laterally, whereas in the central part all motor neurons expressed GFP. Caudally, GFP was expressed differentially, with high expression in the dorso-medial portion, whereas other motor neurons displayed low expression (Fig. 1C,D). Subtypes of hypoglossal motor neurons are topographically organized in conserved and stereotyped positions according to the tongue muscles they innervate (Aldes, 1995; McClung and Goldberg, 2002). The distribution of *Tshz1*-expressing motor neurons indicates that they innervate the extrinsic genioglossus, the palatossus or the hyoglossus retrusor muscles, and the intrinsic inferior and/or superior longitudinalis muscles, but not the intrinsic verticalis and transversus protrusor muscles (Aldes, 1995; Dobbins and Feldman, 1995; McClung and Goldberg, 1999).

In the spinal cord, *Tshz1*-driven GFP was expressed in subpopulations of Isl1/2^+^, ChAT^+^ motor neurons. In particular, we detected GFP expression in the area where the phrenic motor column develops already at E10.5, and GFP expression persisted in the phrenic motor column throughout fetal development (Fig. 1E; Fig. S1B). Phrenic motor neurons innervate the diaphragm and express the transcription factor Pou3f1 in addition to the pan-motor neuron markers Isl1/2 and ChAT (Machado et al., 2014; Philippidou et al., 2012). GFP was expressed in 85% of phrenic motor neurons at E12.5 and P0.5 (Fig. 1E,F). In addition, *Tshz1*-driven GFP expression was transiently detected in motor neurons in the brachial and lumbar spinal cord (Fig. 1G; Fig. S2B). Low GFP levels were present in some differentiating motor neurons (Olig2^+^, Isl1/2^+^), whereas the majority of early postmitotic motor neurons (Olig2^−^, Isl1/2^+^) expressed GFP (Fig. S2A,B). In the lumbar spinal cord, GFP persisted until E15.5 but was undetectable at P0.5 (Fig. 1G,H; data not shown). Next, we defined identities of brachial and lumbar spinal motor neuronal subtypes that expressed *Tshz1* at E12.5 (Fig. S2D-O). GFP was detected in about two-thirds of Foxp1^+^ neurons in the lateral and medial parts of the lateral motor columns (LMCl and LMCm) at E12.5, but only in about 10% of neurons in the medial motor column (MMC; Isl1/2^+^, Lhx3^+^: Fig. S2D-G,L-O). In the thoracic spinal cord, GFP was expressed at a low level in about 5% of preganglionic motor neurons (PGCs; Foxp1^+^, Isl1/2^+^) and about 10% of hypaxial motor neurons (HMCs: Isl1/2^+^, Lhx3^−^; Fig. S2H-K). In summary, persistent high levels of *Tshz1*-driven GFP were observed in hypoglossal and phrenic motor neurons, and only transient GFP expression in other subsets of motor neurons (Fig. 1I). We did not detect Tshz1-driven GFP in GATA3^+^ or Chx10 (Vsx2)^+^ V2 interneurons, or in Sim1*^+^* V3 interneurons at E12.5 (Fig. S3A,B). The related protein Tshz3 is expressed in the nervous system (Caubit et al., 2005, 2010). Tshz3 protein was not present in hypoglossal (Caubit et al., 2010) or phrenic motor neurons at E12.5, but was expressed in cells of the lateral and medial motor columns in the lumbar spinal cord (Fig. S3C,D).

### *Tshz1* expression in motor neurons is necessary for the survival of newborn mice

**Fig. 2.**
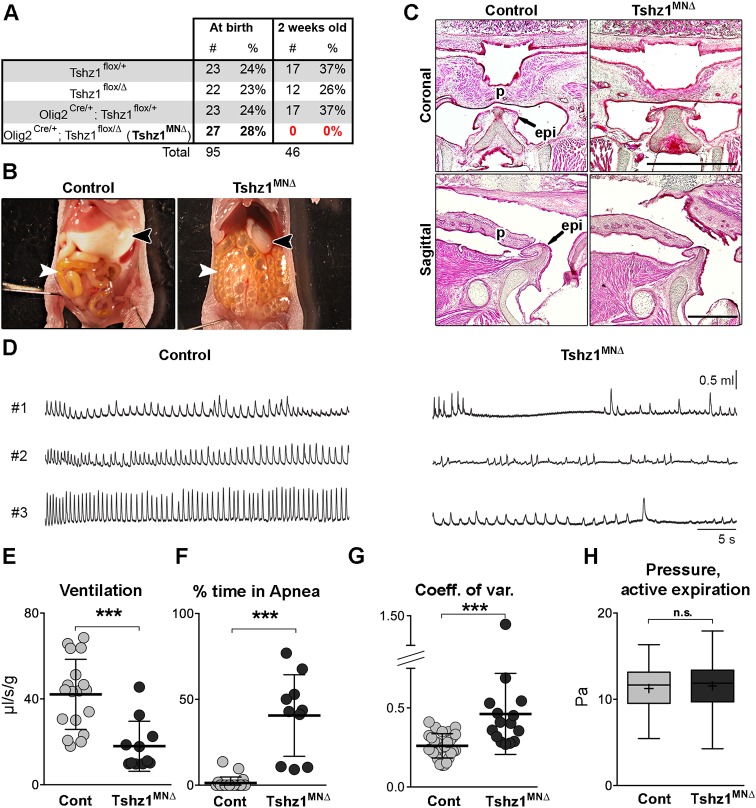
***Tshz1^MN^*^Δ^ mice (P0.5) do not feed, display aerophagia and respiratory distress.** (A) Proportions of offspring with different genotypes obtained after heterozygous mating (*Olig2^Cre^; Tshz1*^Δ*/+*^×*Tshz1^flox^^/flox^*). Note that the *Tshz1^MN^*^Δ^ mutants were born at a normal Mendelian ratio but died during the early postnatal period with none surviving by 2 weeks after birth. (B) Comparison of the abdominal cavity of control and *Tshz1^MN^*^Δ^ pups at P0.5. Note that milk in the stomach was not detected in *Tshz1^MN^*^Δ^ pups (black arrowhead), but their intestine was filled with air (white arrowhead). (C) Histological analysis of the oral cavity of control and *Tshz1^MN^*^Δ^ pups. Note that the palate (p) and epiglottis (epi) were normally formed. (D) Examples of plethysmographic tracks of three control and three *Tshz1^MN^*^Δ^ mutant pups at P0.5. (E) Quantification of ventilation (V_E_, defined as V_t_/T_tot_) of control and *Tshz1^MN^*^Δ^ pups. (F) Quantification of time in apnea (>2 s without a breath) of control and *Tshz1^MN^*^Δ^ pups. (G) Coefficient of variation of the breathing frequencies (calculated as s.d./mean) in *Tshz1^MN^*^Δ^ and control mice. (H) Active expiration in newborn mice is observed during vocalization; pressure during active expiration was determined from plethysmographic recordings of control and *Tshz1^MN^*^Δ^ mutant pups during vocal breathing. Pa, Pascal. Box plots represent the first and third quartile; the line is the median and the plus symbol is the mean value; whiskers represent minimum and maximum. Scale bars: 50 µm. At least three animals/genotype were analyzed. Each dot in E-G represents one animal, bars represent the mean±s.d. Unpaired *t*-test: ***P<0.001. n.s., non-significant.

This expression pattern suggested a possible role for *Tshz1* during specification of distinct motor neuronal subtypes. We conditionally mutated *Tshz1* in the ventral motor neuron progenitor domain using *Olig2^Cre^* (*Olig2^Cre^; Tshz1*^Δ*/Flox*^; hereafter called *Tshz1^MN^*^Δ^). It should be noted that *Tshz1* is not expressed in V2 or V3 interneurons that are also generated from progenitors recombined by *Olig2^Cre^* (see Fig. S3A,B). *Tshz1^MN^*^Δ^ mutant animals were born at a normal Mendelian ratio but did not survive beyond P0.5 (Fig. 2A). We noted that *Tshz1^MN^*^Δ^ pups were unable to feed and had no milk in their stomach. Dissection of the abdominal cavity revealed that their intestines were filled with air (aerophagia; Fig. 2B). In addition, *Tshz1^MN^*^Δ^ pups displayed signs of respiratory distress and cyanosis. These phenotypes are similar, if not identical, to those observed in *Tshz1* null mutant mice (*Tshz1*^Δ*/*Δ^, referred as *Tshz1^NULL^*; Coré et al., 2007). *Tshz1^NULL^* mice display oral cavity malformations, i.e. absence of soft palate and flattened epiglottis. As expected for an *Olig2^Cre^*-specific mutation, we did not observe such changes in the soft palate or epiglottis of *Tshz1^MN^*^Δ^ mice at P0.5 (Fig. 2C). We conclude that aerophagia and feeding deficits in *Tshz1^MN^*^Δ^ pups are caused by a neuronal dysfunction.

Next, we quantified the breathing deficits in *Tshz1^MN^*^Δ^ animals at P0.5 using plethysmographic recordings (Fig. 2D). Compared with controls, *Tshz1^MN^*^Δ^ mutant mice displayed longer breath duration (T_tot_; Fig. S4A) and smaller tidal volume (V_t_; Fig. S4B). Therefore, ventilation (V_E_; V_t_/T_tot_), representing the amount of air that reaches the lungs per minute, was reduced by half (Fig. 2E). In addition, *Tshz1^MN^*^Δ^ mutants spent more time in apnea, which was defined as a period longer than 2 s without a breath (Fig. 2F), although the length of individual apneic periods was similar in *Tshz1^MN^*^Δ^ mutants and control mice (Fig. S4D). The coefficient of variation of the breathing frequency (calculated as s.d./mean) was higher in *Tshz1^MN^*^Δ^ mutants than in controls, highlighting the irregular breathing of *Tshz1^MN^*^Δ^ animals (Fig. 2G). We observed that the expiratory period (defined as the time between the end of inspiration and onset of the next inspiration cycle) was prolonged in *Tshz1^MN^*^Δ^ animals (Fig. S4C). During normal breathing, expiration of newborn mice is passive and relies on the relaxation of the rib cage/lungs, indicating that the longer expiratory period might be due to less frequent inspiration events. To exclude expiratory deficits, we also analyzed expiration during vocalization, as vocal bursts depend on active expiration (Hernandez-Miranda et al., 2017). In *Tshz1^MN^*^Δ^ mutants, active vocal expirations were normal in strength and frequency (Fig. 2H; Fig. S4E,F). Furthermore, we did not detect breaths that were abnormally short, apneustic-like prolonged inspirations, or the presence of forced expiratory efforts that would suggest impaired inspiratory/expiratory timing as a cause of apneas. Therefore, we conclude that *Tshz1^MN^*^Δ^ mutants present with a reduced inspiratory motor drive, which results in shallow breathing, an increased incidence of apneas and rhythm irregularities.

### *Tshz1* is specifically required for hypoglossal and phrenic motor neuron survival

**Fig. 3.**
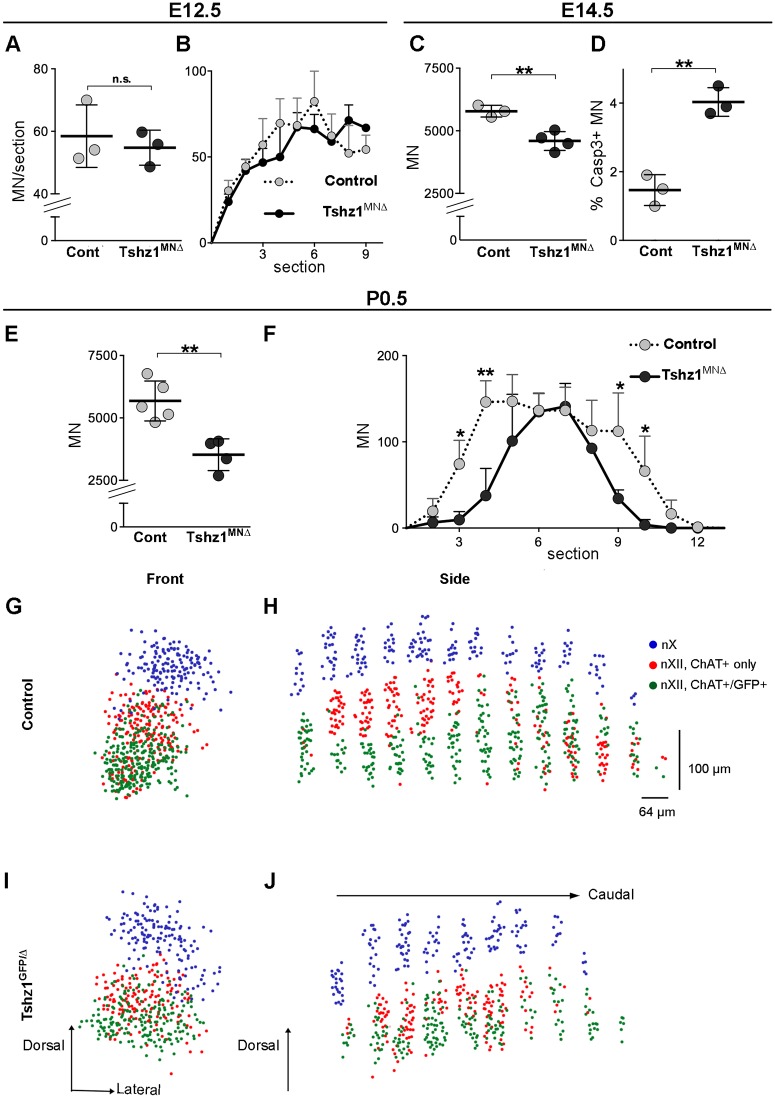
**Progressive loss of hypoglossal motor neurons in *Tshz1^MN^*^Δ^ mice.** (A,B) Numbers of hypoglossal motor neurons (Isl1/2^+^) at E12.5 in *Tshz1^MN^*^Δ^ and control embryos shown as the average number of motor neurons per section (A), and neuron numbers along the rostro-caudal axis in nine consecutive sections starting from the rostral end of the nucleus (B). (C) Total numbers of hypoglossal motor neurons (Isl1/2^+^) at E14.5 in *Tshz1^MN^*^Δ^ and control embryos. (D) Percentage of apoptotic hypoglossal motor neurons (cleaved Caspase3^+^, Isl1/2^+^) in *Tshz1^MN^*^Δ^ and control animals at E14.5. (E) Total numbers of hypoglossal motor neurons (ChAT^+^) in *Tshz1^MN^*^Δ^ and control mice at P0.5. (F) Counts per section of neurons in the hypoglossal nucleus along the antero-posterior axis of control and *Tshz1^MN^*^Δ^ mice at P0.5. Note that the length of the nucleus was reduced in *Tshz1^MN^*^Δ^ mice. (G-J) 3D reconstruction of the hypoglossal nucleus of *Tshz1^GFP/+^* and *Tshz1^GFP/^*^Δ^ mice at P0.5. Blue: motor neurons of the adjacent dorsal nucleus of the vagus (nX). Red: motor neurons of the hypoglossal nucleus that do not express GFP. Green: motor neurons of the hypoglossal nucleus that express GFP. The numbers of Tshz1-negative (i.e. ChAT^+^/GFP^−^) neurons were not significantly changed (253±55 and 193± 42 in control and *Tshz1^MN^*^Δ^ mice, respectively; non-significant), but the numbers of ChAT^+^/GFP^+^ neurons were reduced (382±60 and 251±41 in control and *Tshz1^MN^*^Δ^ mice, respectively, *P*<0.05). Examples of immunostainings used for the 3D reconstruction are presented in Fig. S5. (G,H) Front and side view of the control hypoglossal nucleus. (I,J) Front and side view of the *Tshz1^MN^*^Δ^ mutant hypoglossal nucleus. At least three animals per condition were analyzed. Each dot in A-E represents one animal, bars represent the mean± s.d. Unpaired *t*-test: n.s., non-significant; *P<0.05; **P<0.01.

Previous studies have demonstrated that tongue innervation by hypoglossal nerves is required for suckling and feeding (Fujita et al., 2006; Fukushima et al., 2014). We hypothesized that impaired development of hypoglossal motor neurons might cause the feeding deficit observed in *Tshz1^MN^*^Δ^ mice. To assess this, we counted hypoglossal motor neurons at different developmental stages (Fig. 3A-F). Motor neurons are generated in excess and up to 50% undergo apoptosis during a critical period from E13.5 to E14.5 (Dekkers et al., 2013; Yamamoto and Henderson, 1999). Similar numbers of hypoglossal motor neurons were present in *Tshz1^MN^*^Δ^ and control animals at E12.5 (Fig. 3A,B). We observed a 20% loss of hypoglossal motor neurons at E14.5 (Fig. 3C), which became more pronounced by P0.5 when the loss amounted to 38% (Fig. 3E). The loss of motor neurons was due to increased apoptosis detected by staining for cleaved-caspase 3 (Fig. 3D). 3D reconstructions showed the hypoglossal nucleus was shortened along the antero-posterior axis in *Tshz1^GFP/^*^Δ^ mutants (Fig. 3F). However, the overall nuclear organization was conserved and GFP-expressing motor neurons were located at the same relative positions (Fig. 3G-J; Fig. S5). Altogether, our data demonstrate that in *Tshz1^MN^*^Δ^ mice, hypoglossal motor neurons are generated in normal numbers but their survival is impaired.

**Fig. 4.**
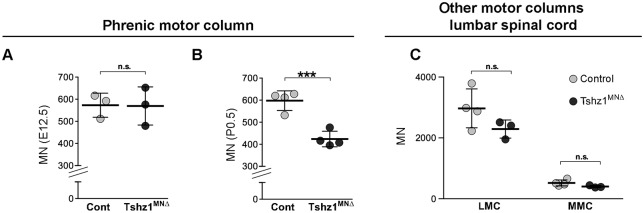
**Progressive loss of phrenic but not other spinal motor neurons in *Tshz1^MN^*^Δ^ mice.** Phrenic motor neurons were identified in the cervical spinal cord using Pou3f1 and Isl1/2 (E12.5) or ChAT (P0.5) antibodies. (A) Total number of phrenic motor neurons at E12.5 in *Tshz1^MN^*^Δ^ and control embryos. (B) Total number of phrenic motor neurons at P0.5 in *Tshz1^MN^*^Δ^ and control embryos. (C) Motor neurons of the lateral and medial motor columns (LMC and MMC) in the lumbar spinal cord were identified using an anti-ChAT antibody and quantified in control and *Tshz1^MN^*^Δ^ mice at P0.5. At least three animals per condition were analyzed. Each dot represents one animal, bars represent the mean±s.d. Unpaired *t*-test: n.s., non-significant; ***P<0.001.

Because *Tshz1^MN^*^Δ^ animals displayed severe breathing deficits, we analyzed phrenic motor neurons, which innervate the diaphragm. Phrenic motor neurons can be distinguished from other motor neurons at the same axial level (C3-C5) by their location between the medial and hypaxial motor columns and by co-expression of Isl1/2 and Pou3f1. The number of phrenic motor neurons was unchanged at E12.5 but reduced at P0.5 in *Tshz1^MN^*^Δ^ mice (Fig. 4A,B). Thus, phrenic motor neurons were generated in correct numbers but subsequently died. Genes specifically expressed in phrenic but not in other types of ventral motor neurons have been previously identified (Machado et al., 2014; Philippidou et al., 2012). We analyzed the expression of a subset of these phrenic-specific genes in *Tshz1^MN^*^Δ^ mutants at E12.5, i.e. at a stage before motor neuron loss was observed. Expression of *Alcam*, *Pou3f1*, *Hoxa5* and *Hoxc5* was correctly initiated in phrenic motor neurons of *Tshz1^MN^*^Δ^ mice, indicating that phrenic motor neurons retained their identity (Fig. S6). *Tshz1* was also transiently expressed in other spinal motor neurons. However, in the lateral and medial motor columns, we detected no loss of ChAT^+^ motor neurons compared with controls in *Tshz1^MN^*^Δ^ mice at P0.5 (Fig. 4C).

### Tshz1 mutation impairs hypoglossal and phrenic nerve innervation

**Fig. 5.**
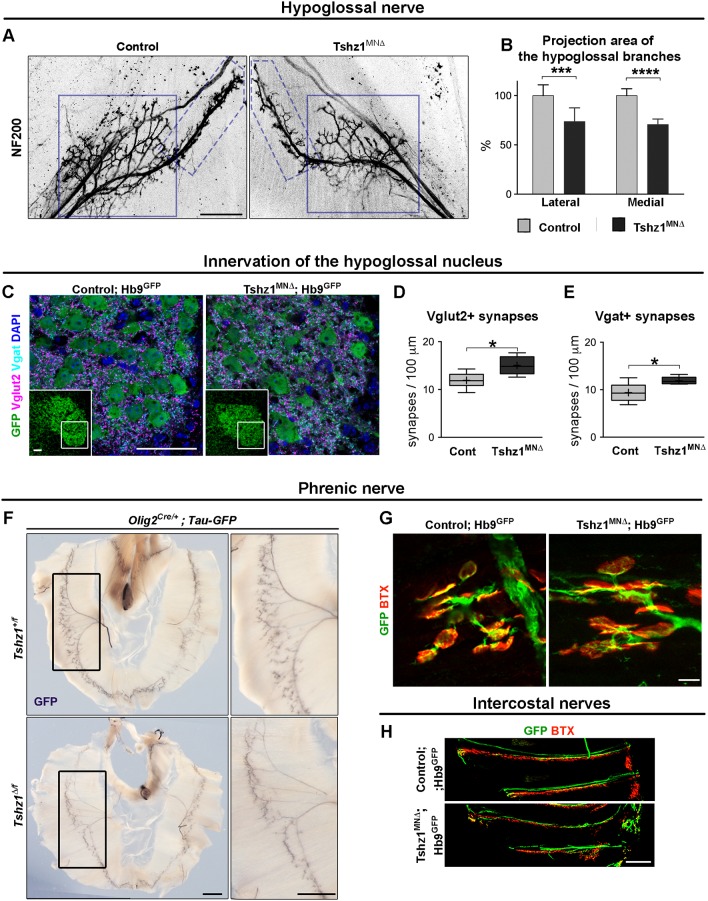
**Changes in hypoglossal and phrenic nerve thickness and hypoglossal innervation pattern in newborn *Tshz1^MN^*^Δ^ mice.** (A) Appearance of the hypoglossal nerve visualized at P0.5 by whole-mount staining using antibodies against NF200. The lateral and medial branches of the hypoglossal nerve are outlined by blue and dashed blue boxes, respectively. (B) Quantification of the fraction of the innervation area covered by NF200 signals, i.e. the areas occupied by the hypoglossal nerve and its medial and lateral branches. (C) Excitatory and inhibitory innervation of the hypoglossal nucleus (GFP^+^ in *Hb9^GFP^* mice) are visualized using antibodies against Vglut2 (magenta) and Vgat (cyan), respectively. The area of the hypoglossal nucleus analyzed is indicated in the insets. (D) Quantification of Vglut2^+^ synapses per 100 µm of soma perimeter of GFP^+^ hypoglossal motor neurons in *Hb9^GFP^* mice. (E) Quantification of Vgat^+^ synapses per 100 µm of GFP^+^ hypoglossal soma perimeter in *Hb9^GFP^* mice. (F) Visualization of phrenic nerves using GFP antibodies on whole-mount diaphragm preparations. Images on the right are magnifications of the boxed areas on the left. (G) Immunostaining of GFP^+^ axons and AChR using Alexa Fluor 555-conjugated α-bungarotoxin (BTX) to visualize neuromuscular junctions in the diaphragm muscle of *Hb9^GFP^* mice. (H) Immunohistological analysis of whole-mount preparations of intercostal muscles of control and *Tshz1^MN^*^Δ^ P0.5 mice; axons labeled by GFP and acetylcholine receptor clusters were visualized using anti-GFP antibodies (green) and conjugated α-bungarotoxin (BTX, red) in *Hb9^GFP^* mice. In D,E, boxes indicate first quartile, median and third quartile; whiskers indicate minimum and maximum; mean is marked by a plus symbol. Scale bars: 200 µm (A); 50 µm (C); 1 mm (F, left); 100 µm (F, right); 20 µm (G); 500 µm (H). At least three animals per condition were analyzed (five or six medial or lateral branches from three or four animals were analyzed). Data are represented as mean±s.d. Unpaired *t*-test: n.s., non-significant; *P<0.05; ***P<0.001; ****P<0.0001.

Next, we investigated the morphology of the hypoglossal and phrenic nerves at P0.5 (Fig. 5). Innervation of the tongue by the hypoglossal nerve was assessed using antibodies against heavy neurofilament protein (NF200) on whole-mount tongue preparations (Fig. 5A). The overall innervation pattern of the hypoglossal nerve was conserved (one ventral and one medial branch that separate inside the tongue), but the nerve was thinner and less branched in *Tshz1^MN^*^Δ^ than in control mice. We analyzed the overall projections of medial and lateral branches of the hypoglossal nerve and observed a 25-30% reduction in the projection area of both branches in *Tshz1^MN^*^Δ^ mice (Fig. 5B; Fig. S7). We also analyzed excitatory and inhibitory innervation of hypoglossal motor neurons using antibodies directed against Vglut2 (Slc17a6) and Vgat (Slc32a1) proteins, which mark presynaptic terminals (Fig. 5C). Quantification of the density of Vglut2 and Vgat punctae revealed an increase of both inhibitory and excitatory synapses onto hypoglossal motor neurons in the *Tshz1^MN^*^Δ^ compared with control mice (Fig. 5D,E). This indicates that premotor neurons increase their connectivity to those motor neurons that are spared from cell death in the *Tshz1^MN^*^Δ^ animals. We conclude that innervation deficits correlate with the impaired feeding and aerophagia of *Tshz1^MN^*^Δ^ mice.

Phrenic nerve innervation of the diaphragm was visualized using either membrane-bound GFP expressed upon recombination with *Olig2^Cre^* (Fig. 5F; *Tau-LSL-mGFP*; Hippenmeyer et al., 2005) or a *Hb9^GFP^* transgene expressed in ventral motor neurons (Fig. 5G,H; Wichterle et al., 2002). Whole-mount preparations showed that the diaphragm of *Tshz1^MN^*^Δ^ mice was innervated and that the overall innervation pattern appeared to be correct (Fig. 5F). However, phrenic nerves were thinner in *Tshz1^MN^*^Δ^ mice (Fig. 5F). Neuromuscular junctions assessed by α-bungarotoxin labeling were correctly formed in *Tshz1^MN^*^Δ^ mutants (Fig. 5G). Finally, innervation of thoracic muscle was unaffected (Fig. 5H). We propose that a deficit of the phrenic motor column accounts for the impaired breathing of *Tshz1^MN^*^Δ^ mice.

### Tshz1 does not affect respiratory rhythm generation but impairs the pattern of motor bursts

**Fig. 6.**
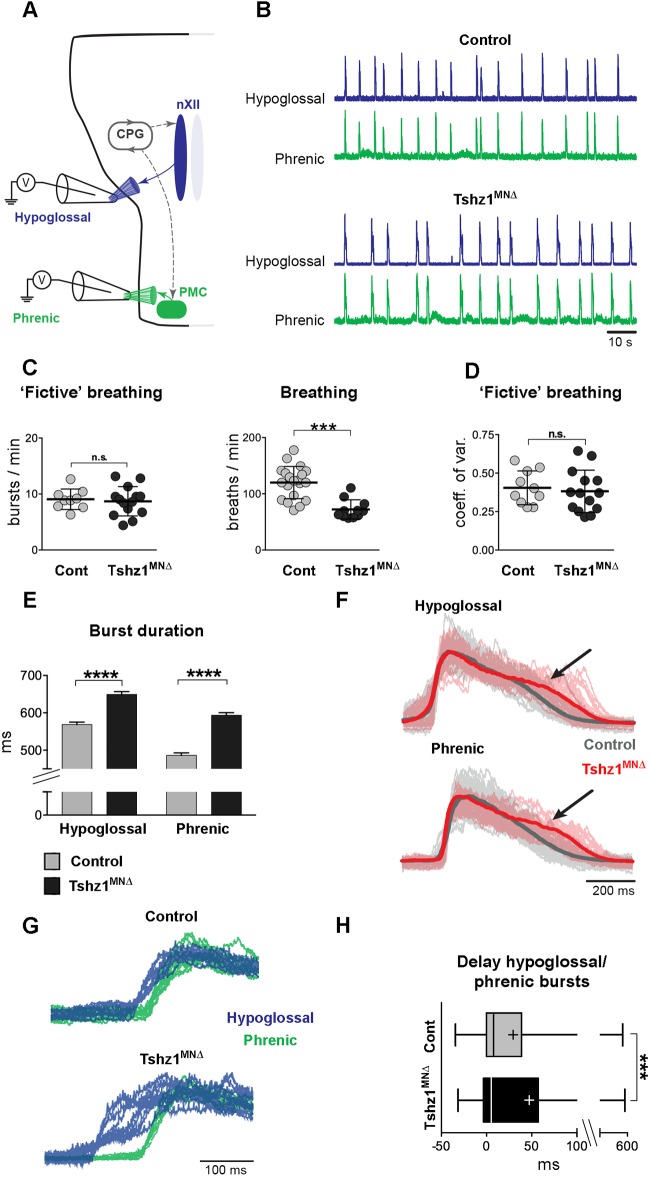
**Hypoglossal and phrenic nerves are functional but the coordination of their burst patterns is changed in *Tshz1^MN^*^Δ^ mice.** (A) Scheme showing setup used for electrophysiology analysis at E18.5; hypoglossal and phrenic nerves are indicated. nXII, hypoglossal nucleus (blue); CPG, central pattern generator (gray); PMC, phrenic motor column (green). (B) Representative examples of hypoglossal and phrenic nerve electrophysiological activity from *Tshz1^MN^*^Δ^ and control mice. Traces illustrate neurograms. (C) Plots of ‘fictive’ (left, bursts/min of phrenic nerve *in vitro* preparation) and ‘real’ (right, breaths/min in plethysmographic recording) respiratory frequency. (D) Coefficient of variation for ‘fictive’ breathing frequencies (calculated as s.d./mean) in *Tshz1^MN^*^Δ^ and control mice. (E) Burst duration of spontaneous phrenic and hypoglossal nerve activities. (F) Superimposition of hypoglossal (top) and phrenic (bottom) spontaneous bursts of motor activity measured in the controls (gray, *n*=60 bursts) and *Tshz1^MN^*^Δ^ mutants (red, *n*=40 bursts) displayed in B. The thicker lines represent the average motor burst pattern; traces are aligned at the onset of the motor burst. Note that *Tshz1^MN^*^Δ^ animals show a prolonged decay time compared with control animals as indicated by the black arrows. (G) Superimposition of the initiation of hypoglossal (blue) and phrenic (green) motor bursts measured in controls (upper panel, *n*=10 bursts) and *Tshz1^MN^*^Δ^ mutants (lower panel, *n*=10 bursts). (H) Phrenic bursts initiate a few milliseconds after the hypoglossal bursts. This delay in control and *Tshz1^MN^*^Δ^ mice is represented as a boxplot with whiskers (min to max). Lines and plus signs represent the median and the mean, respectively. Boxes represent first and third quartile. At least eight animals/genotype were analyzed. Graphs in C-E show mean±s.d. Fictive breathing and the coefficient of variation *in vitro* showed Gaussian distributions and were statistically analyzed using unpaired *t*-test: n.s., non-significant. The coefficient of variation *in vivo*, burst frequencies, burst duration and delays did not show Gaussian distributions and were analyzed using the Kruskal–Wallis test followed by Dunn's multiple comparison test: ***P<0.001; ****P<0.0001.

Breathing and swallowing are highly automated behaviors driven by central pattern generators located in the hindbrain (Del Negro et al., 2018). Phrenic and hypoglossal nerves burst synchronously but not simultaneously during the inspiratory phase of breathing, i.e. hypoglossal activity precedes phrenic activity. Hypoglossal activity results in tongue muscle contraction and expansion of the upper airways, which decrease airway resistance in anticipation of the inspiratory effort (Ghali, 2015). This synchronization is also necessary for feeding in order to avoid maladaptive outcomes such as the swallowing of air or airway blockage (Moore et al., 2014). We tested whether respiratory rhythm generation and coordinated phrenic/hypoglossal motor activity were affected in *Tshz1^MN^*^Δ^ mutants. For this, we used E18.5 isolated brainstem-spinal cord preparations, which retain a fictive breathing behavior, to record spontaneous hypoglossal and phrenic nerve activity *in vitro* (Fig. 6A,B). In contrast to the reduction of the frequency of breaths in *Tshz1^MN^*^Δ^ animals (*in vivo* plethysmographic recordings), burst frequencies of the phrenic nerve and regularity of the rhythm (assessed with the coefficient of variation) were similar in *Tshz1^MN^*^Δ^ and control mice (Fig. 6C,D). Further, activities of the phrenic and hypoglossal nerves remained synchronous in preparations of *Tshz1^MN^*^Δ^ animals (Fig. 6B). Careful examination of hypoglossal and phrenic bursts revealed that the total duration of hypoglossal and phrenic motor bursts was increased in *Tshz1^MN^*^Δ^ animals (Fig. 6E), due to a lengthening of the burst decay (Fig. 6F). In addition, the temporal coordination between the onsets of hypoglossal and phrenic motor activity was altered *in vitro*, as hypoglossal nerves fired earlier in *Tshz1^MN^*^Δ^ mutants than in control animals (Fig. 6G,H). Altogether, these data indicate that the irregular breathing of *Tshz1^MN^*^Δ^ mutants *in vivo* is not explained by deficits in rhythm generation. However, changes in the coordination of hypoglossal and phrenic activity might underlie or contribute to the observed deficits in breathing and feeding in *Tshz1^MN^*^Δ^ mutants.

### Preventing apoptosis does not rescue the impaired survival of *Tshz1^MN^*^Δ^ mutants

**Fig. 7.**
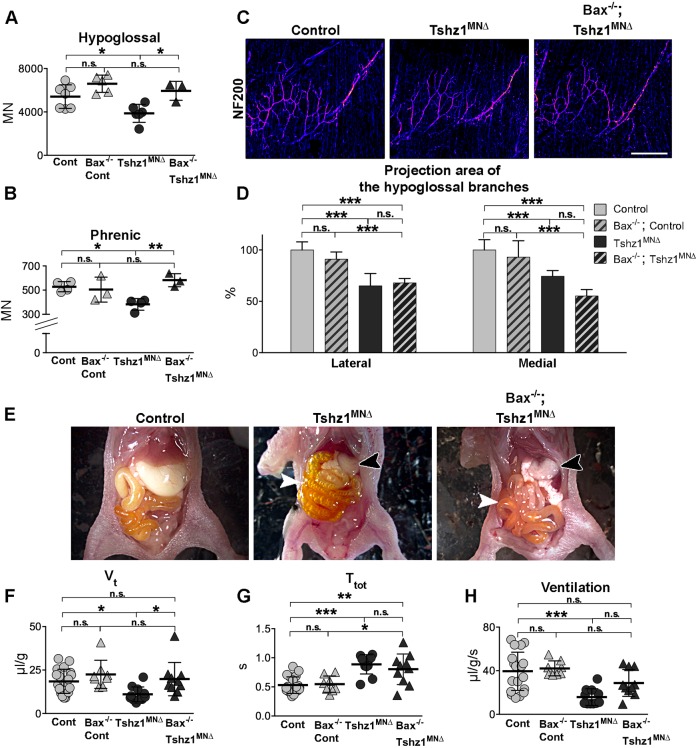
**The additional *Bax* mutation fails to fully rescue feeding and breathing deficits in newborn *Tshz1^MN^*^Δ^ mice.** (A,B) Total numbers of motor neurons at P0.5 in the hypoglossal nucleus (A) and phrenic motor column (B) after introduction of the *Bax* mutation. (C) Appearance of the lateral and medial branches of the hypoglossal nerve of *Bax^−/−^; Tshz1^MN^*^Δ^ mice and littermates visualized at P0.5 by whole-mount staining using antibodies against NF200. NF200 signal is visualized using the ‘fire’ LUT plugin on ImageJ in which the intensity of the signal is indicated by a blue-to-red (weak to strong signal) color code. (D) Quantification of the fraction of the innervation area covered by NF200 signals, i.e. the areas occupied by the hypoglossal nerve and its medial and lateral branches. (E) Abdominal cavity of *Bax^−/−^ ; Tshz1^MN^*^Δ^ mice and littermates. Black arrowheads point to stomach, white arrowheads to intestines. (F) Tidal volume (V_t_) calculated from plethysmography recordings of *Bax^−/−^; Tshz1^MN^*^Δ^ and littermate mice. (G) Breath duration (T_tot_). (H) Ventilation (V_t_/T_tot_). Scale bar: 200 µm. At least three animals per condition were analyzed in C,D. Each dot represents one animal in A,B,F-H, bars represent the mean±s.d. Ordinary one-way ANOVA with Tukey's multiple comparison test: n.s., non-significant; *P<0.05; **P<0.01; ***P<0.001.

Mutation of the pro-apoptotic gene *Bax* is commonly used to rescue apoptosis in neurons, but the elimination of *Bax* cannot rescue additional functional deficits (Hua et al., 2013; Philippidou et al., 2012; Tanaka et al., 2014; White et al., 1998). We tested whether the mutation of *Bax* could rescue the cell death and physiological phenotypes observed in *Tshz1^MN^*^Δ^ animals (*Bax^−/−^; Tshz1^MN^*^Δ^). The mutation of *Bax* was sufficient to rescue the loss of motor neurons, and no difference in the composition of the hypoglossal nucleus or phrenic motor column was observed in *Bax^−/−^; Tshz1^MN^*^Δ^ and *Bax^−/−^* animals at P0.5 (Fig. 7A,B). Interestingly, preventing apoptosis did not rescue deficits in hypoglossal innervation or the impaired survival of *Tshz1^MN^*^Δ^ pups (Fig. 7C-E). In particular, *Bax^−/−^; Tshz1^MN^*^Δ^ pups were still unable to feed although they did not display air in their intestine (Fig. 7E). Plethysmographic analysis demonstrated that breathing parameters improved but were not fully restored in *Bax^−/−^; Tshz1^MN^*^Δ^ animals (Fig. 7F-H). Specifically, the tidal volume was rescued, but breath duration did not recover, resulting in an incomplete rescue of ventilation. Thus, further motor neuron dysfunction(s) in addition to cell death cause the respiratory distress and inability to feed.

## DISCUSSION

A common transcriptional program (*Isl1/2^+^*, *Hb9^+^*) defines the generic identity of ventral motor neurons. These neurons subsequently diversify and express subtype-specific genes that correlate with the muscle they innervate. We show here that Tshz1, a zinc-finger and homeodomain factor, is persistently expressed in developing hypoglossal and phrenic motor neurons and is essential for their survival and function. We investigated the role of Tshz1 in ventral motor neurons using conditional mutagenesis. In such mice, hypoglossal and phrenic motor neurons are born in correct numbers, but many die between E13.5 and E14.5. Severe feeding and breathing problems accompany this developmental deficit. The deficiency in neuronal survival can be rescued by the elimination of the pro-apoptotic factor *Bax*, but this does not suffice to fully rescue the dysfunction of phrenic and hypoglossal motor neurons. Thus, even when the motor neuron numbers are rescued, *Bax^−/−^; Tshz1^MN^*^Δ^ mutants cannot feed, and display deficits in breathing and reduced innervation of hypoglossal nerve targets. Thus, Tshz1 coordinates the survival and function of phrenic and hypoglossal motor neurons.

### Genes that control hypoglossal and phrenic motor neuron formation

We show here that *Tshz1* specifically functions during development of phrenic and hypoglossal motor neurons. However, *Tshz1* is also expressed in other motor neuron populations, such as those contained within the lateral motor column, but these neurons express *Tshz1* transiently and their generation is not affected by *Tshz1* mutation. Thus, the action of *Tshz1* is axially restricted to the caudal hindbrain and cervical spinal cord. The anterior-posterior identity of motor neurons depends on Hox genes expressed in a spatially restricted manner in the hindbrain and spinal cord. At a given axial level, Hox activity is refined in motor neurons by subtype-specific co-factors such as Foxp1 in the lateral motor columns (Dasen and Jessell, 2009). In *Drosophila*, the protein Tsh, homolog of Tshz1, has been shown to directly interact with the Hox protein Sex combs reduced (Scr) to promote trunk structure development and repress head formation (de Zulueta et al., 1994; Fasano et al., 1991; Röder et al., 1992). This raises the possibility that Hox genes cooperate with *Tshz1* during the specification of hypoglossal and phrenic motor neurons.

In mammals, three Teashirt homologs (*Tshz1-3*) exist and any one of them can replace *Tsh* in *Drosophila* (Manfroid et al., 2004). Interestingly, *Tshz1* and *Tshz3* are co-expressed in the lateral motor column but not in hypoglossal or phrenic motor neurons (Fig. S3C; Caubit et al., 2010). Thus, the specificity of Tshz1 function in phrenic and hypoglossal motor neurons could reflect the absence of redundantly acting Teashirt factors in these motor neuronal subtypes. It is interesting to note that Tshz3 was previously shown to control survival of motor neurons in the nucleus ambiguus, i.e. a pool of brainstem neurons that innervate the upper airways (Caubit et al., 2008). Thus, both *Tshz1* and *Tshz3* can take over essential roles in development of motor neuronal subtypes.

### Physiological consequences of *Tshz1* mutations

Hypoglossal and phrenic motor pools control movement of the tongue and the diaphragm, respectively, and are specifically affected by *Tshz1* mutations. In particular, *Tshz1^MN^*^Δ^ mutants have feeding problems that we assign to a hypoglossal dysfunction. Suckling behavior and milk intake depend on the contraction of tongue muscles and surgical impairment of hypoglossal nerves prevents feeding in rats (Fujita et al., 2006; Fukushima et al., 2014; Lowe, 1980; McLoon and Andrade, 2013). The medial and lateral branches of the hypoglossal nerve innervate protrusive and retrusive tongue muscles, respectively, and the projection pattern of both branches was impaired in *Tshz1^MN^*^Δ^ mutants. Tongue protruder muscles are required to keep the airways open (Edwards and White, 2011; Fregosi, 2011; McLoon and Andrade, 2013). In contrast, tongue retrusors are needed during swallowing and are inactive during normal breathing (Fujita et al., 2006; Fukushima et al., 2014; McLoon and Andrade, 2013). The observed changes in innervation of hypoglossal nerve targets might thus affect breathing in addition to feeding behavior.

*Tshz1^MN^*^Δ^ mice also show long periods of apnea and breathe more shallowly. In addition, *Tshz1^MN^*^Δ^ mice display an unusual phenotype, an accumulation of air in the intestine. Aerophagia in newborn mice has been previously observed in *Sim2*, *Lgr5* and *Tshz1^NULL^* mutants (Coré et al., 2007; Morita et al., 2004; Shamblott et al., 2002). Aerophagia in *Sim2* and *Tshz1^NULL^* mutant mice was assigned to cleft palate and other malformations of the oral cavity (Coré et al., 2007; Shamblott et al., 2002). In *Lgr5* mutant mice, it was attributed to an abnormal fusion of the tongue to the floor of the oral cavity (Morita et al., 2004). Our analysis relied on a conditional mutation of *Tshz1* in ventral motor neurons, and thus unambiguously shows that a neuronal dysfunction causes aerophagia.

Swallowing interrupts the respiratory cycle, resulting in an apneic period during which the larynx is closed, and the bolus moves through the pharynx into the esophagus (McFarland and Lund, 1993; Paydarfar et al., 1995). A disruption of this process might cause aerophagia. Aerophagia is also observed in humans where it is frequently assigned to abdomino-phrenic dyssynergia, a condition characterized by a deficit in abdominal and diaphragm muscle coordination (Dike et al., 2017; Ercoli et al., 2018; Villoria et al., 2011). We, therefore, propose that the phrenic dysfunction is responsible for breathing deficits and also contributes to the aerophagia.

The specificity of Tshz1 function in a small subset of motor neurons indicates that this factor determines a program unique for phrenic and hypoglossal neurons rather than a generic motor neuron transcriptional program. Whereas the innervation pattern of the tongue was disrupted, diaphragm muscle innervation was largely preserved in *Tshz1^MN^*^Δ^ mice despite the severe consequences of the mutation on breathing behavior. Changes in premotor input that resulted in uncoordinated movement have been defined in locomotor circuits (Baek et al., 2017; Hinckley et al., 2015; Song et al., 2016). However, the neuronal circuitries that control complex orofacial behaviors such as swallowing and breathing are incompletely characterized and only phrenic premotor neurons but not hypoglossal ones have been defined (Wu et al., 2017). To test whether the overall synaptic input that shapes hypoglossal activity is altered in *Tshz1^MN^*^Δ^ mutants, we counted the density of synapses onto hypoglossal motor neurons. We detected an increase in the number of excitatory and inhibitory synapses onto the remaining hypoglossal motor neurons in *Tshz1^MN^*^Δ^ mutants. This indicates that the premotor neurons still recognize their correct targets and compensate for the neuronal loss in *Tshz1^MN^*^Δ^ mutants by more densely innervating the remaining motor neurons. However, many neuronal cell types synapse onto motor neurons to regulate their activity, and we cannot exclude the possibility that deficits in innervation by specific subtypes contribute to the behavioral deficits in *Tshz1^MN^*^Δ^ mutant mice.

Genetic mutation of the pro-apoptotic gene *Bax* is commonly used to rescue apoptosis in the central nervous system (Hua et al., 2013; Philippidou et al., 2012; Tanaka et al., 2014; White et al., 1998). However, the *Bax* mutation cannot rescue associated functional deficits. For instance, the *Hoxa5/c5* mutation in mice causes a loss of phrenic motor neurons accompanied by a lack of diaphragm innervation, and an additional *Bax* mutation rescues neuronal numbers but not innervation (Philippidou et al., 2012). We show here that the additional mutation of *Bax* in a *Tshz1^MN^*^Δ^ background rescued hypoglossal and phrenic motor neuron numbers but did not rescue hypoglossal innervation deficits or survival of the mutant animals. Thus, feeding and breathing behaviors are still impaired in *Bax^−/−^; Tshz1^MN^*^Δ^ double mutant mice. We conclude that the deficits caused by the mutation of *Tshz1* result not only in a loss of motor neurons, but also in functional deficits such as the changed coordination of phrenic and hypoglossal nerve activity. Thus, the mutation might impact coordinated movements of the tongue and of the diaphragm, thereby impairing the vital ability to coordinate orofacial muscles during respiration and swallowing.

## MATERIALS AND METHODS

### Mouse strains

Mouse strains carrying *Olig2^Cre^* (Dessaud et al., 2007), *Tshz1^Flox^*, *Tshz1*^Δ^ and *Tshz1^GFP^* (Ragancokova et al., 2014) alleles have been described. *Bax* mutant (Knudson et al., 1995) and *Rosa26-Tomato Ai14^Flox^* (Madisen et al., 2010) strains were obtained from The Jackson Laboratory. All experiments were performed in accordance with the guidelines and policies of the European Union, the Max Delbrück Center for Molecular Medicine and the Institut des Neurosciences Paris-Saclay.

### Histology, immunofluorescence, *in situ* hybridization and quantification

Hematoxylin-Eosin staining was performed on cryosections (12-18 µm) using Weigert's iron Hematoxylin (Sigma-Aldrich) and Eosin Y. Immunofluorescence analyses were performed on cryosections or on whole-mount diaphragm (Lin et al., 2001) using the following primary antibodies: rat anti-GFP (1:1500, Nacalai Tesque, GF090R), mouse anti-Isl1/2 [1:400, Developmental Studies Hybridoma Bank (DSHB), 39.4D5 and 40.2D6], guinea pig anti-Isl1/2 (1:5000, a kind gift from T. Jessell and Susan Brenner-Morton, Columbia-University, New York, USA), rabbit anti-Foxp1 (1:500, Abcam, ab16645), mouse anti-Lhx3 (1:300, DSHB, 67.4E12), goat anti-ChAT (1:300, Millipore, AB144P), rabbit anti-Olig2 (1:500, Millipore, AB9610), rabbit anti-cleaved caspase3 (1:300, Cell Signaling, 5A1E), guinea pig anti-Tshz1 (1:4000; Ragancokova et al., 2014), rabbit anti-RFP (1:1000, Biotrend, 600-401-379), rabbit anti-Pou3f1/Oct6 (1:500, Abcam, ab126746), guinea pig anti-VGLUT2 (1:500, Synaptic Systems, 135404), rabbit anti-VGAT (1:500, Synaptic Systems, 131002), rabbit anti-GATA3 (1:500, Abcam, ab106625) and sheep anti-Chx10 (1:300, Abcam, ab16141) Secondary antibodies coupled either with Alexa Fluor 555, Alexa Fluor 488 or Alexa Fluor 639 were used at 1:500 (Molecular Probes). For whole-mount diaphragm staining, Alexa Fluor 488-conjugated α-bungarotoxin (1:1000, Molecular Probes) was used. Images were acquired on a Zeiss LSM 700 confocal microscope. Chromogenic and immunofluorescent *in situ* hybridization were performed as described (Wildner et al., 2013). All probes were synthesized using an RNA labeling kit (Roche) as specified by the manufacturer. Probe for *Sim1* was a gift from Martyn Goulding (Salk Institute, San Diego, USA). Probe for *Tshz1* was amplified from total brain cDNA.

Motor neuronal subtypes were identified according to their location in the hindbrain and spinal cord, and by their expression of specific transcription factors. Hypoglossal motor neurons close to the midline in the caudal hindbrain (rhombomeres 7/8) were identified with Isl1/2 (E12.5) or ChAT (P0.5) antibodies. At E14.5 and P0.5, motor neurons in the entire nucleus were counted; at E12.5, only nine consecutive sections of the rostral half of the nucleus were analyzed. Phrenic motor neurons are located at cervical levels between the medial and lateral motor columns and specifically express *Pou3f1*. Motor pools were quantified on serial sections, using at least three animals per genotype. Motor neurons were counted unilaterally on every sixth section for characterization of *Tshz1* expression, and bilaterally on every fourth section for *Tshz1^MN^*^Δ^ mice analyses.

Images used for synapse quantification were acquired on a Leica SP8 microscope with a HC PL APO CS2 63.0×1.30 Glycerol objective. Stacks of nine or ten images were recorded at 330 nm increments. Laser power and settings were identical for all samples in an experiment. The recorded image stacks were deconvolved using Leica Lightning processing in LASX. Automated calculation of perisomatic bouton density was performed offline in MatLab (MathWorks) using the Image Processing Toolbox and custom-written routines. Briefly, image de-noising with edge preservation was performed using Guided Filtering. The GFP channel was then binarized using a locally adaptive threshold. Small processes were pruned by eroding the masked areas, removing small objects, and then dilating the remaining masked areas. A perisomatic sample region was defined within 6 pixels (∼400 nm) of the masked soma. Labeled boutons were binarized using Otsu's methods. Masked boutons that were found to reside within the sample region were counted and the synapse density was calculated as the number of counted boutons divided by the soma perimeter.

### 3D reconstruction

Cryosections of *Tshz1^GFP/+^* or *Tshz1^GFP/^*^Δ^ hindbrains at P0.5 were stained with Isl1/2 and GFP antibodies. Motor neurons of the vagus motor nucleus were used as a landmark. In the hypoglossal nucleus, motor neurons were either Isl1/2^+^, GFP^−^ or Isl1/2^+^, GFP^+^. To allocate Cartesian coordinates to the motor neurons, we acquired their positions using the spots function in Imaris software (bitplane). Their *xy* positions were recalculated using the midline as the *y*-axis, and the lowest point of the hypoglossal nucleus as the origin. The z-axis coordinate depended on the section: *z*=0 corresponded to the most rostral section with hypoglossal MNs. Subsequent sections were separated by 64 µm. Coordinates were exported to ‘R’ and the 3D reconstruction was made using a previously described script (Dewitz et al., 2018).

### Whole-mount staining

Whole-mount staining of diaphragm was adapted from Lin et al. (2000). Briefly, trunks of P0.5 animals were fixed in 4% paraformaldehyde for 4 h at 4°C. After fixation, diaphragms were dissected, incubated in 0.1 M glycine in PBS, rinsed and permeabilized in 0.5% Triton X-100 in PBS. Diaphragms were blocked for at least 90 min in dilution buffer (500 mM NaCl, 3% bovine serum albumin, 5% goat serum in 0.5% Triton X-100 in PBS), and incubated with primary antibodies overnight at 4°C. After three washes in 0.5% Triton X-100 in PBS, preparations were incubated with secondary antibodies and Alexa Fluor 488-conjugated α-bungarotoxin (1:1000, Molecular Probes) for 2 h. After washes, diaphragms were flat-mounted on slides and pictured. Diaphragm innervation patterns are variable even between controls (Laskowski et al., 1991), which precluded a quantitative assessment.

Whole-mount staining of the tongue was carried out after P0.5 preparations of tongue and lower jaw were fixed for 4 h in 4% paraformaldehyde, bleached in Dent's Bleach (10% H_2_O_2_, 13.3% DMSO, 53.3% methanol) for 24 h and re-fixed in Dent's fixative (20% DMSO, 80% methanol) for 24 h. The preparations were stained for 5 days with chicken anti-NF200 antibody in blocking buffer [0.5% of normal goat serum (Invitrogen), 20% DMSO, 75% PBS, 0.025% sodium azide] and then incubated for 2 days with Alexa Fluor 555-coupled secondary antibody in blocking buffer. After washing, samples were dehydrated in methanol and cleared in BABB (1/3 benzyl alcohol. 2/3 benzyl benzoate). Preparations were placed in a homemade glass chamber filled with BABB. Images were acquired as *z*-stacks using a Zeiss LSM700 confocal microscope. For the analysis of the innervation of hypoglossal targets, we used a *z*-projection method with maximal intensity to define the area of the projections (ImageJ 1.42). The fraction of the innervation area covered by NF200 signals, i.e. the hypoglossal nerve and its medial and lateral branches, was quantified. A double-blind approach was applied to calculate the signal-occupied area.

### Plethysmography

Breathing of pups was recorded 3-6 h after birth as described (Bouvier et al., 2010; Hernandez-Miranda et al., 2017). Briefly, freely moving P0 animals were placed in an airtight plethysmographic chamber of 20 ml volume connected to a differential pressure transducer (Validyne DP 103-14) and a reference chamber. Variation of pressures was sampled at 1 kHz and digitalized using a Labmaster interface. Breathing was monitored for 5 min. Injection of 2.5 µl of air into the chamber with a Hamilton syringe was used as a calibration and allowed the quantification of several breathing parameters. For quantification, apnea-free periods of more than 30 s were used. Breath duration (T_tot_), tidal volume (V_t_) and apnea duration, defined as ventilation arrest longer than 2 s, were obtained from analyses of plethysmographic traces with Elphy software (developed by Gerard Sadoc, UNIC, CNRS, France, https://www.unic.cnrs-gif.fr/software.html). Tidal volume (V_t_) was normalized to the animal weight and used to calculate ventilation (V_E_=V_t_/T_tot_ in µl/s/g).

### Electrophysiology

Electrophysiological recordings of hypoglossal and phrenic nerves were performed as described (Ruffault et al., 2015). Briefly, E18.5 animals were delivered by caesarian section, anesthetized on ice and dissected on ice-cold oxygenated artificial cerebrospinal fluid (aCSF) containing 128 mM NaCl, 8 mM KCl, 1.5 mM CaCl_2_, 1 mM MgSO_4_, 24 mM NaHCO_3_, 0.5 mM Na_2_HPO_4_, 30 mM glucose, pH 7.4. Brainstem-spinal cord preparations were obtained after transverse sections through the ponto-medullary region and the C6 spinal cervical segment. Preparations were transferred to the recording chamber (4 ml) and perfused with oxygenated aCSF at 30°C. After 30 min of recovery, hypoglossal and phrenic nerves were recorded using extracellular bipolar suction electrodes (A-M Systems). During the recordings, nerves were perfused with normal aCSF. Raw signals were amplified (1000×) and filtered (0.3-30 kHz) through a high-gain AC amplifier (7P511, Grass Instrument). Signals were then integrated (time constant 50 ms, Neurolog System, Digitimer), digitalized at 10 kHz (Digidata A322A), recorded and analyzed using pClamp9 software (Molecular Devices). To determine the delay between hypoglossal and phrenic activities, we defined the starting point of the burst as 15% of the burst maximum amplitude. Thus, the initial ‘slow’ slope effect was not included in the quantification.

### Statistical analyses

All statistical analyses were performed with GraphPad Prism 6 software. Normality of the data was tested with d'Agostino-Pearson omnibus normality test. For comparing two datasets, we used unpaired two-tailed *t*-test. For multiple datasets, we used ordinary one-way ANOVA with Tukey's multiple comparisons test. Burst durations, coefficient of variation (*in vivo*) and nerve delays did not follow a Gaussian distribution; therefore, we used Kruskal–Wallis test followed by Dunn's multiple comparisons test.

## Supplementary Material

Supplementary information
