# Supplementary material for: Teashirt 1 (Tshz1) is essential for the development, survival and function of hypoglossal and phrenic motor neurons in mouse
Source: Development. 2019 Sep 6;146(17):dev174045. doi: 10.1242/dev.174045 (PMC6765129; doi:10.1242/dev.174045)
Supplement: Supplementary information [file develop-146-174045-s1.pdf]

Supplementary Figures

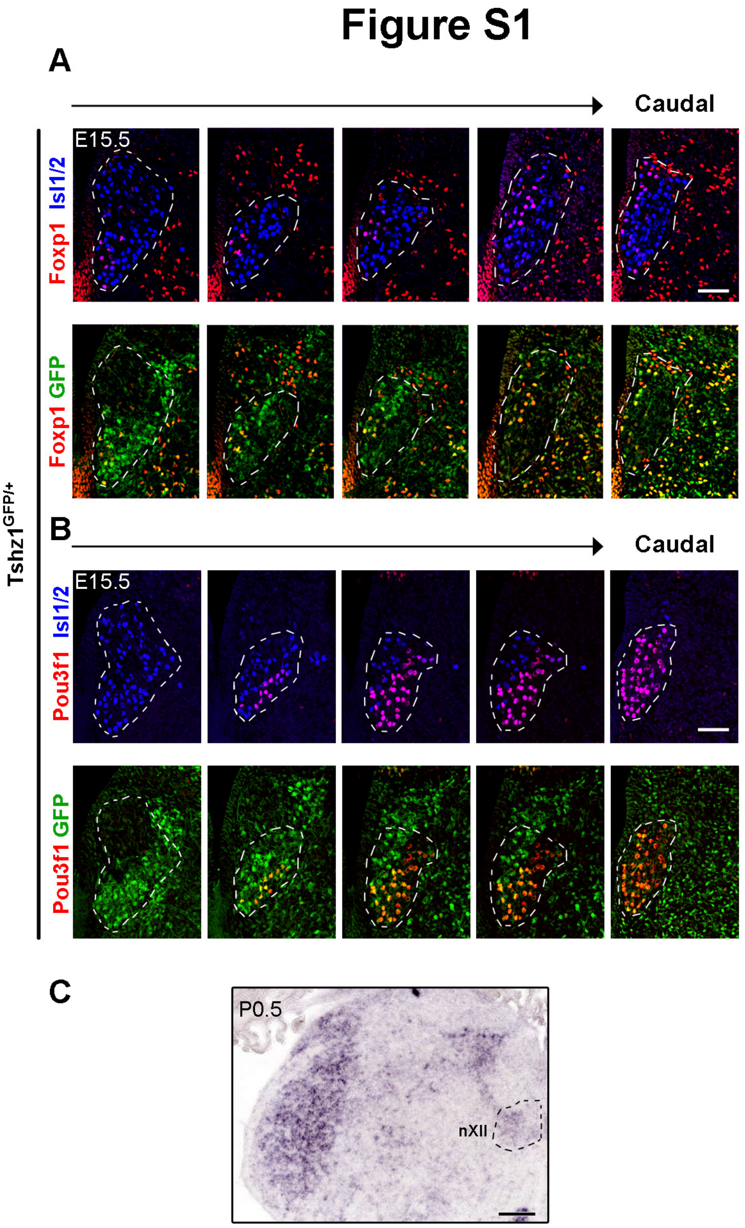

Figure S1: Co-expression of *Tshz1* with Foxp1 and Pou3f1 in the hypoglossal nucleus

(A) Immunohistochemical analysis of the hypoglossal nucleus of control mice (E15.5) with antibodies against Isl1/2, GFP and Foxp1 on sections from different levels along the rostro-caudal axis. The hypoglossal nucleus is outlined (white dashed line) (B) Immunohistochemical analysis of control mice (E15.5) with antibodies against Isl1/2, GFP and Pou3f1 on sections from different levels along the rostro-caudal axis. The hypoglossal nucleus is outlined (white dashed line) (C) *In situ* hybridization for *Tshz1* at P0.5. The hypoglossal nucleus is outlined (black dashed line). Note that *Tshz1* mRNA is still detected in the hypoglossal nucleus (nXII). Scale bar in (A, B): 50 µm, in (C): 200 µm. 3 animals/genotype were analyzed.

Development • Supplementary information

Figure S2

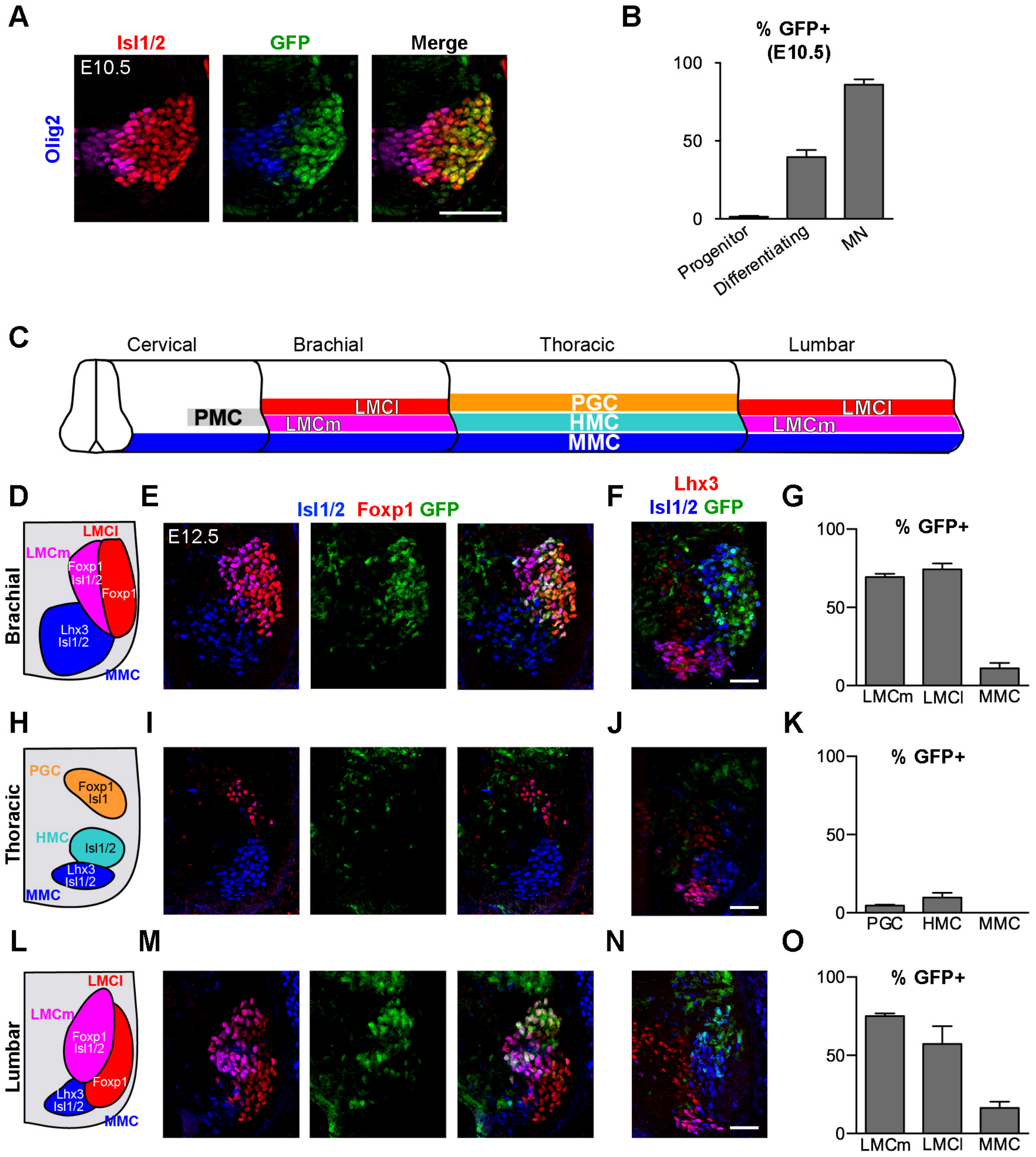

Figure S2: Differential expression of *Tshz1* in motor columns of the spinal cord

For the analysis of Tshz1 expression, we used ***Tshz1***$^{GFP/+}$ animals and assessed GFP expression˙ (A) Immunohistochemistry at E10.5 with antibodies against Isl1/2, GFP and Olig2 in the lumbar spinal cord. (B) Quantification of progenitors (Olig2+, Isl1/2-), differentiating motor neurons (Olig2+, Isl1/2+) and motor neurons (Isl1/2+,

Development • Supplementary information

Olig2-) co-expressing GFP at E10.5. (C) Scheme of different motor neuron populations in the spinal cord. PMC, phrenic motor column; MMC, medial motor column; LMCl/m, lateral motor column lateral/medial; PGC, preganglionic chain; HMC, hypaxial motor column. (D-O) At E12.5, motor neuron populations were analyzed by immunohistochemistry at different levels of the spinal cord. (D,H,L) Schemes of different motor columns and their molecular markers on transverse spinal cord sections. (E,I,M) Immunohistochemistry using antibodies against Isl1/2, Foxp1 and GFP. (F,J,N) Immunohistochemistry using antibodies against Isl1/2, Lhx3 and GFP. (G,K,O) Percentage of motor neuron subtypes expressing GFP in brachial, thoracic and lumbar spinal cord. Scale bars: 50 µm. 3 animals/genotype were analyzed. Data are represented as mean ± S.D.

Figure S3

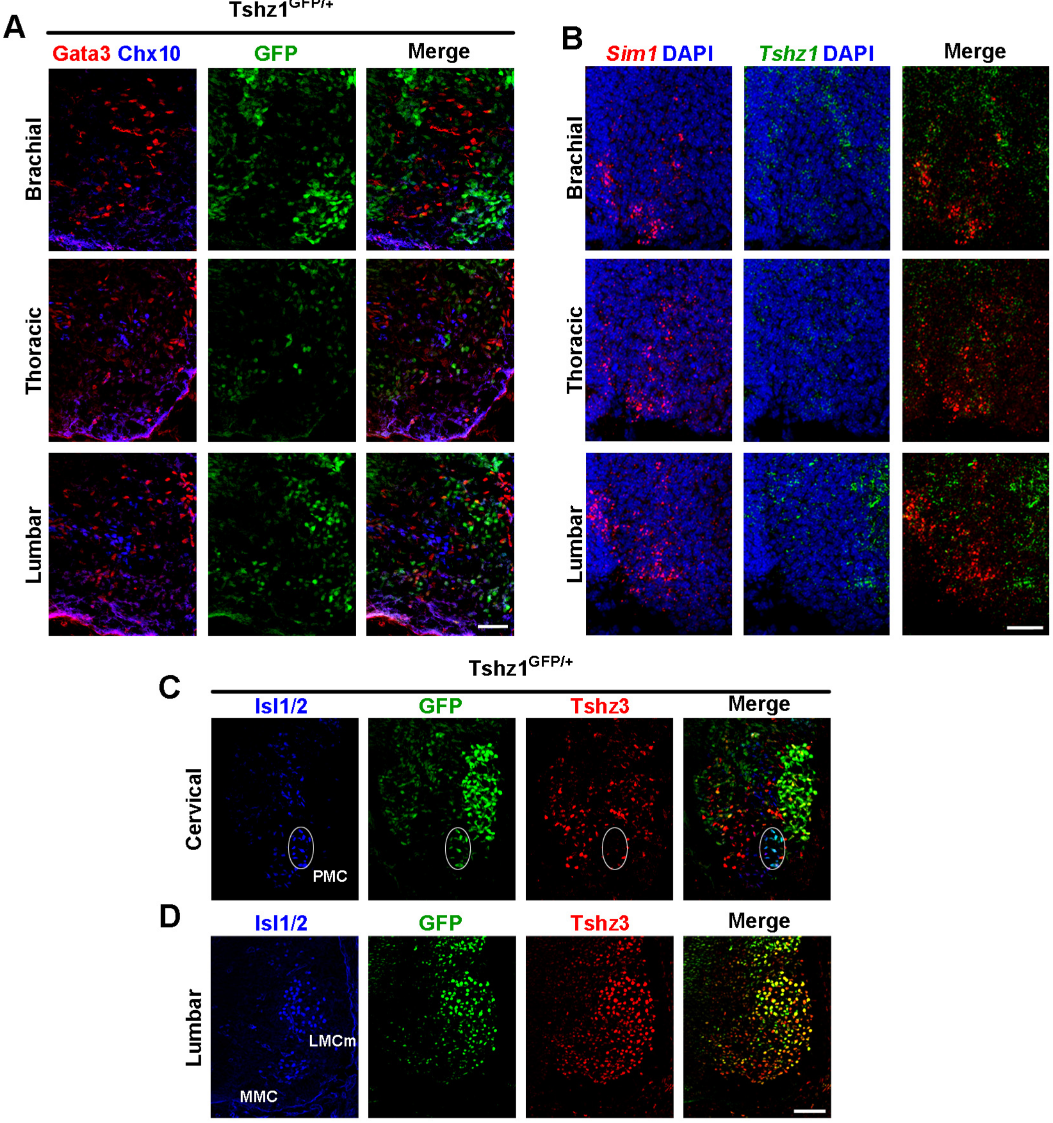

Figure S3: Expression of Tshz1 and Tshz3 in different neuronal populations of the spinal cord at E12.5

(A) Immunohistochemistry at E12.5 with antibodies against GFP, Gata3 and Chx10 in the brachial, thoracic and lumbar spinal cord of *Tshz1*^{GFP} mice. Note that GFP is not expressed in V2a (Chx10+) and V2b (Gata3+) neurons. (B) Double fluorescence *in situ* hybridization using *Tshz1* and *Sim1* riboprobes, demonstrating that *Tshz1* is not expressed in V3 (*Sim1*+) neurons. (C,D) Immunohistochemistry with antibodies against GFP, Tshz3 and Isl1/2 at cervical (C) and lumbar (D) levels of o *Tshz1*^{GFP} mice. Neurons of the phrenic motor column (PMC) are circled in white and do not express Tshz3. LMCm: lateral motor column, medial part; MMC: medial motor column. Scale bars: 50 µm. 3 animals/genotype were analyzed.

Development • Supplementary information

Figure S4

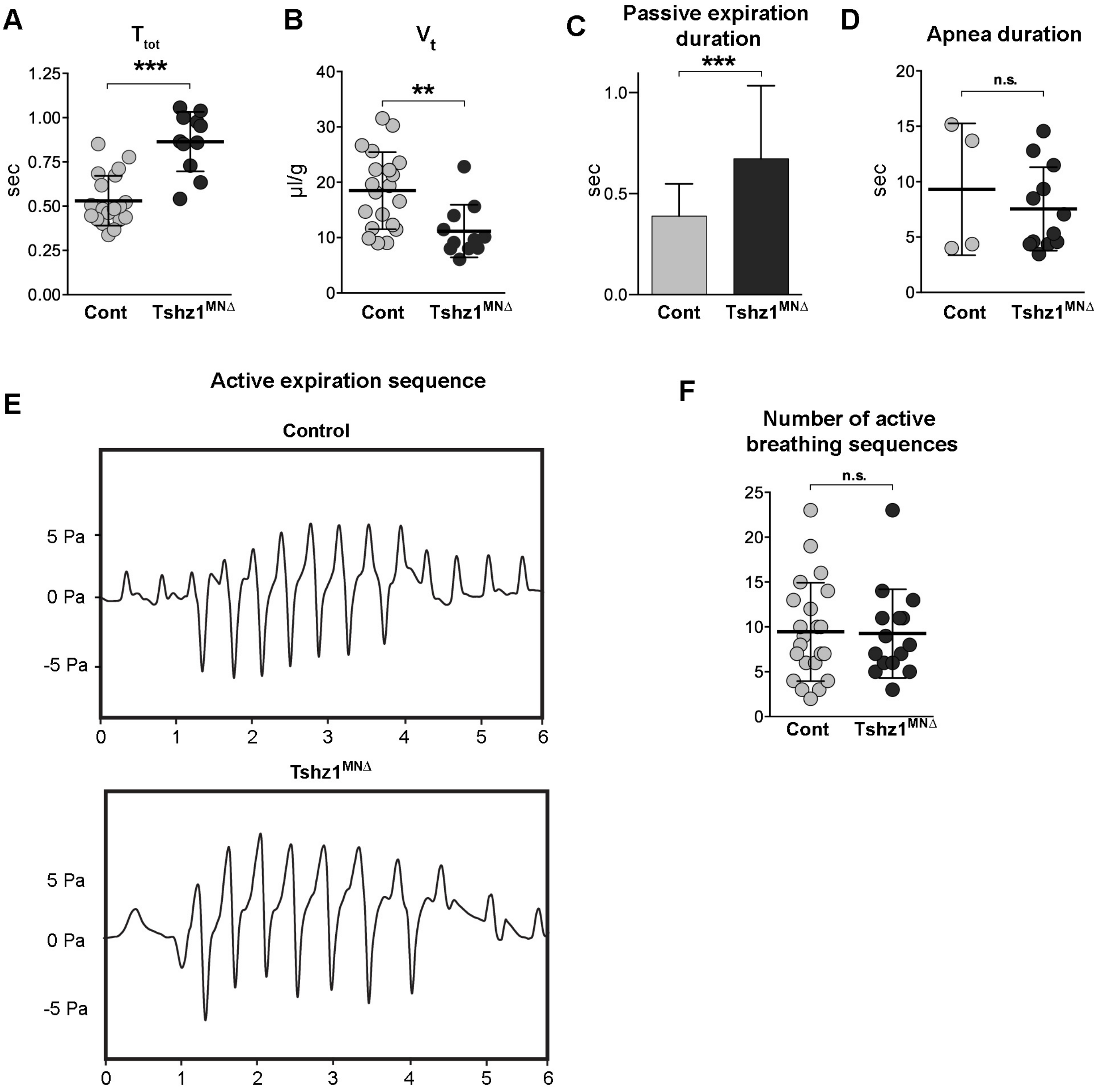

Figure S4: *Tshz1$^{MN\Delta}$* mice display breathing deficits

Plethysmographic recordings showed that several breathing parameters were affected in *Tshz1$^{MN\Delta}$* mice compared to controls. (A) Breathing period (T_{tot}, in seconds). (B) Tidal volume (V_t, in µl/g). (C) Duration of passive expiration period (in seconds); note that the expiratory period is defined as the time between the end of inspiration and onset of the next inspiration cycle, and can thus be caused by

Development • Supplementary information

reduced inspiratory drive. (D) Duration of apneas (in seconds). Note that only 4 control out of 20 animals displayed apneas. (E) Examples of active expiration sequences in control (upper panel) and mutant (lower panel) animals at P0.5. (F) Number of active breathing sequences during plethysmographic recordings over a period of 5 minutes. At least 4 animals/genotype were analyzed. Each dot represents one animal, bars represent mean ± S.D. Unpaired t-test: n.s., non-significant; ***, $p<0.001$; **; $p<0.01$.

Figure S5

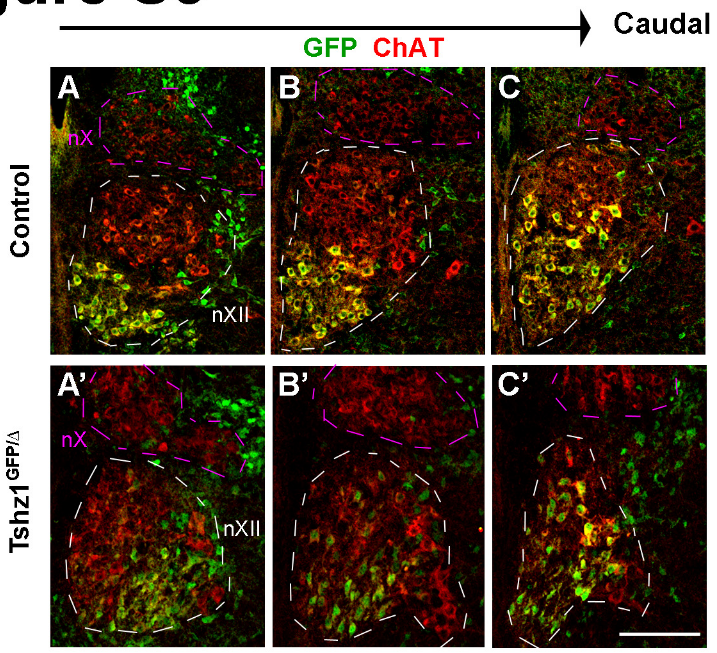

Figure S5: Distribution of GFP+ neurons in the hypoglossal nucleus (P0.5) of control and *Tshz1* mutant mice that carry a *Tshz1^{GFP}* allele

Examples of sections of the hypoglossal nucleus that were used for the 3D reconstruction of the hypoglossal nucleus (see also Fig. 3 G-J). Shown is the hypoglossal nucleus of *Tshz1$^{GFP/+}$* control (A-C) and *Tshz1$^{GFP/\Delta}$* mutant mice (A'-C') at P0.5 stained by antibodies against GFP and ChAT. The hypoglossal (nXII) and dorsal motor nucleus of the vagus (nX) are outlined in white and magenta, respectively. Scale bar: 50 μm.

Figure S6

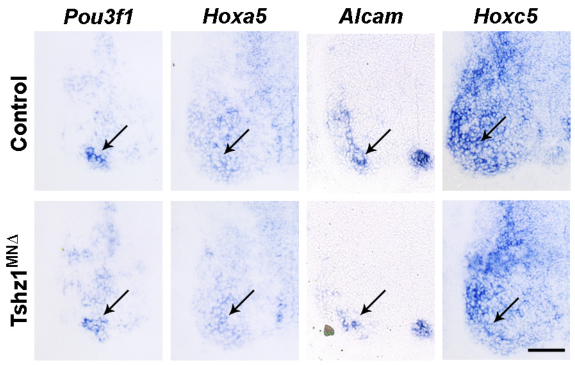

Figure S6: Expression of different phrenic markers in control and *Tshz1*$^{MN\Delta}$ mice at E12.5

In situ hybridization of the phrenic motor nucleus of control and *Tshz1*$^{MN\Delta}$ mice at E12.5 using probes specific for Pou3f1, Hoxa5, Alcam and Hoxc5. The phrenic motor column is indicated by an arrow. Scale bar: 200 µm.

Development • Supplementary information

Figure S7

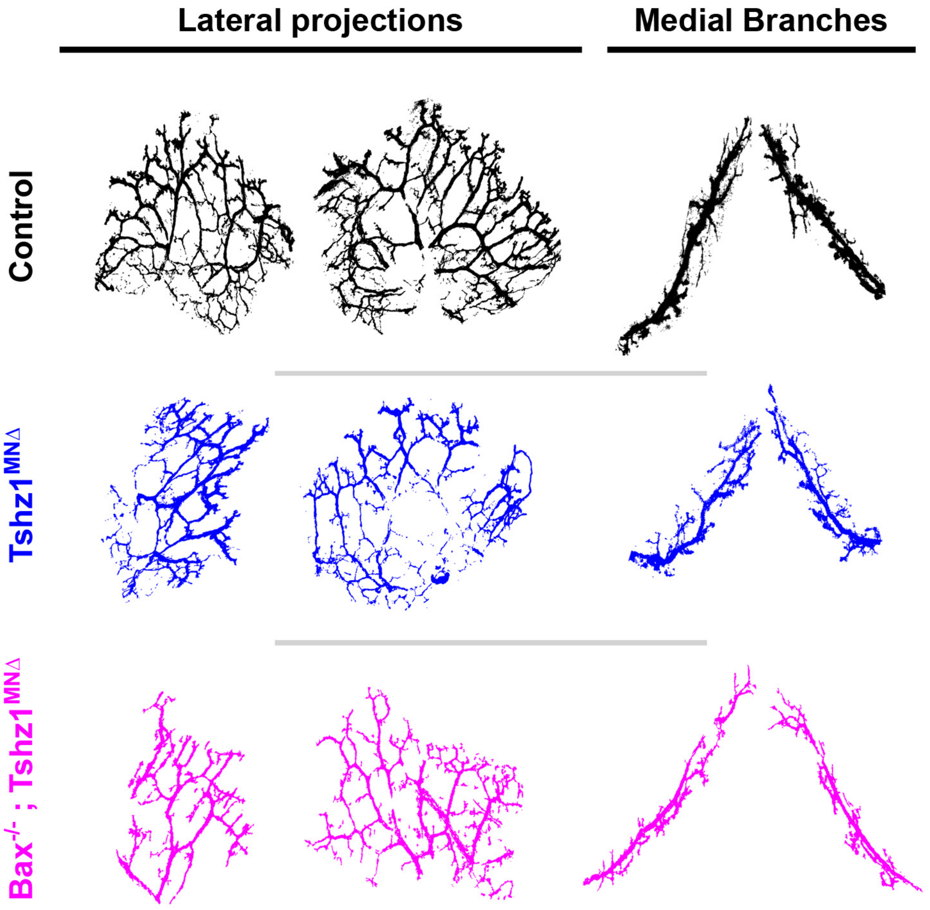

Figure S7: Areas used to calculate the projection surface of the hypoglossal branches

Lateral and medial branches of the hypoglossal nerve in the tongue were analyzed. Shown are images obtained after threshold and ROI adjustment used to calculate the fraction of the innervation area covered by NF200 signals; branches of control (black), *Tshz1*$^{MN\Delta}$ (blue) mice, and *Bax*$^{-/-}$*; Tshz1*$^{MN\Delta}$ mice.

Development • Supplementary information
